# Methods for accounting for neighbourhood self-selection in physical activity and dietary behaviour research: a systematic review

**DOI:** 10.1186/s12966-020-00947-2

**Published:** 2020-04-01

**Authors:** Karen E. Lamb, Lukar E. Thornton, Tania L. King, Kylie Ball, Simon R. White, Rebecca Bentley, Neil T. Coffee, Mark Daniel

**Affiliations:** 1 School of Exercise and Nutrition Sciences, Institute for Physical Activity and Nutrition (IPAN), Deakin University, Geelong, Australia; 2 Clinical Epidemiology and Biostatistics Unit, Murdoch Children’s Research Institute, Melbourne, Australia; 3 Department of Paediatrics, The University of Melbourne, Melbourne, Australia; 4 Centre for Health Equity, Melbourne School of Population and Global Health, The University of Melbourne, Melbourne, Australia; 5 Medical Research Council Biostatistics Unit, University of Cambridge, Cambridge, UK; 6 Health Research Institute, University of Canberra, Canberra, Australia; 7 Department of Medicine, St Vincent’s Hospital, The University of Melbourne, Fitzroy, Victoria Australia

**Keywords:** Bias, Neighbourhood characteristics, Exercise, Diet, Environmental exposure, Adult

## Abstract

**Background:**

Self-selection into residential neighbourhoods is a widely acknowledged, but under-studied problem in research investigating neighbourhood influences on physical activity and diet. Failure to handle neighbourhood self-selection can lead to biased estimates of the association between the neighbourhood environment and behaviour. This means that effects could be over- or under-estimated, both of which have implications for public health policies related to neighbourhood (re)design. Therefore, it is important that methods to deal with neighbourhood self-selection are identified and reviewed. The aim of this review was to assess how neighbourhood self-selection is conceived and accounted for in the literature.

**Methods:**

Articles from a systematic search undertaken in 2017 were included if they examined associations between neighbourhood environment exposures and adult physical activity or dietary behaviour. Exposures could include any objective measurement of the built (e.g., supermarkets), natural (e.g., parks) or social (e.g., crime) environment. Articles had to explicitly state that a given method was used to account for neighbourhood self-selection. The systematic review was registered with the PROSPERO International Prospective Register of Systematic Reviews (number CRD42018083593) and was conducted in accordance with the Preferred Reporting Items for Systematic Reviews and Meta-Analyses (PRISMA) statement.

**Results:**

Of 31 eligible articles, almost all considered physical activity (30/31); few examined diet (2/31). Methods used to address neighbourhood self-selection varied. Most studies (23/31) accounted for items relating to participants’ neighbourhood preferences or reasons for moving to the neighbourhood using multi-variable adjustment in regression models (20/23) or propensity scores (3/23). Of 11 longitudinal studies, three controlled for neighbourhood self-selection as an unmeasured confounder using fixed effects regression.

**Conclusions:**

Most studies accounted for neighbourhood self-selection by adjusting for measured attributes of neighbourhood preference. However, commonly the impact of adjustment could not be assessed. Future studies using adjustment should provide estimates of associations with and without adjustment for self-selection; consider temporality in the measurement of self-selection variables relative to the timing of the environmental exposure and outcome behaviours; and consider the theoretical plausibility of presumed pathways in cross-sectional research where causal direction is impossible to establish.

## Background

Conceptual models suggest that the neighbourhoods in which we live have the potential to affect health-related behaviours such as physical activity and dietary behaviours [1–4]. A growing body of research has investigated links between the local built, natural and social environment on modifiable health-related behaviours [5–7]. While some consistent links have been found (e.g., neighbourhood walkability is associated with active transport [8]), much research on neighbourhood effects on physical activity and dietary behaviours has shown mixed findings [9, 10].

Two challenges in identifying whether environmental features have a causal effect on physical activity and dietary behaviours are that most studies have relied on observational and cross-sectional data. It is rarely ethical or feasible to randomly allocate individuals to live in one neighbourhood or another; the Moving to Opportunity Study in the US being an uncommon example of such an intervention [11]. The preclusion of randomisation is a key obstacle in determining whether observed associations are due to differences in the local environment or other, unmeasured, factors. So, too, does the lack of temporal ordering (possible with longitudinal but not cross-sectional studies) pose challenges for causal inference. A reliance on non-randomised, cross-sectional studies poses serious problems in ruling out a potential impact of neighbourhood self-selection on exposure to particular environment features and health-related behaviours presumed related to these.

**Fig. 1:**
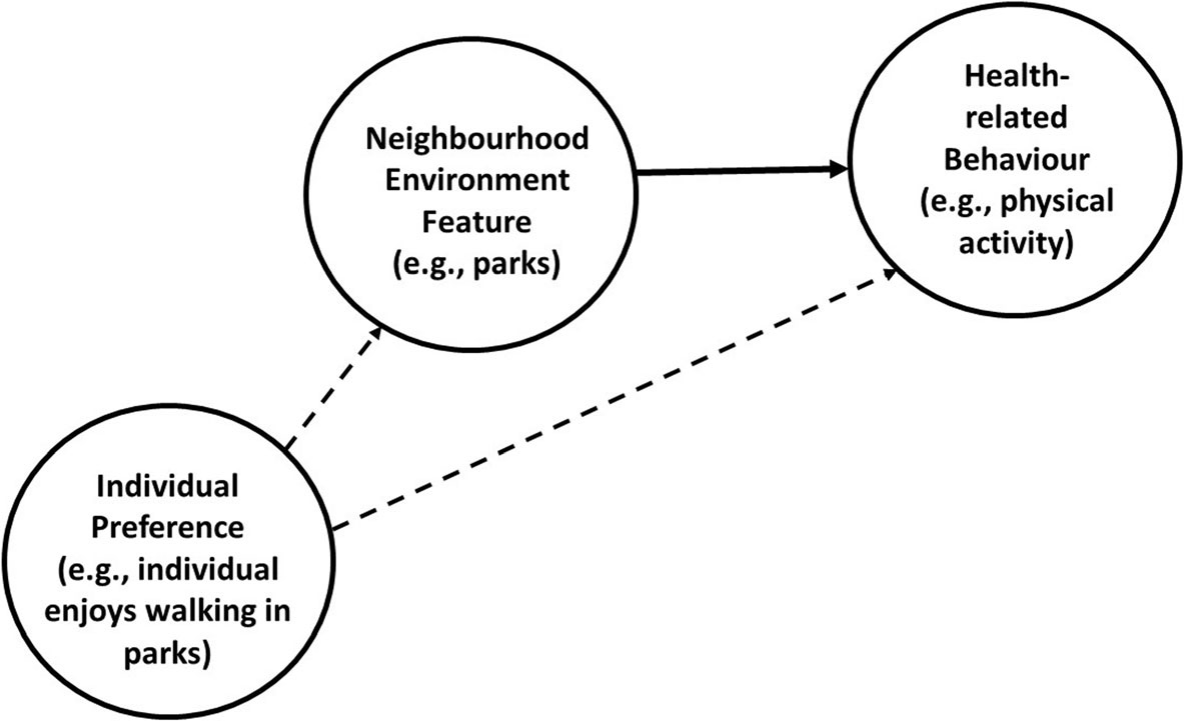
Directed acyclic graph of neighbourhood self-selection as a confounder of the association between a neighbourhood environment feature and a health-related behavioural outcome

Neighbourhood self-selection (also referred to as residential self-selection), refers to people selecting neighbourhoods to live in that have the facilities and resources that suit their preferred lifestyle. If neighbourhood self-selection bias exists, it can be difficult to differentiate the effect of neighbourhood features on behavioural outcomes from the choice to be near features facilitating these preferred behaviours. Consider, for example, the issue of identifying the effect of living near some neighbourhood environment feature, such as parks or sports facilities, on a health-related behavioural outcome, such as physical activity. It could be that individual preference for spending time walking or exercising in parks drives the individual’s selection into a neighbourhood with parks (i.e., the individual “self-selects” into a neighbourhood that supports their preference for walking or exercising in parks). However, this preference also predicts the individual’s health-related behaviour (i.e., their physical activity in this example), as shown in Fig. 1. Therefore, failure to account for this neighbourhood self-selection may bias the estimated effect of the neighbourhood environment feature on the behavioural outcome. This means that the effect could be over- or under-estimated which has implications for public health policies related to neighbourhood (re)design. Neighbourhood self-selection can also occur if people are restricted in their choice of neighbourhood due to the affordability of housing. As discussed by Boone-Heinonen, Gordon-Larsen [12], if lower socioeconomic status neighbourhoods have limited or poor physical activity facilities and residents undertake less physical activity then the relationship between the neighbourhood environment and physical activity could be overestimated. Whilst increasingly acknowledged, neighbourhood self-selection remains an under-studied phenomenon.

Methods for dealing with neighbourhood self-selection have been discussed in physical activity research [12, 13]. In their narrative review, Boone-Heinonen et al. [12] highlighted how longitudinal designs are preferable to enable neighbourhood self-selection to be taken into account in observational research. These designs allow for temporal ordering of exposure and outcome, as well as the possibility of examining within-individual change. In a systematic review of built environment and physical activity associations, McCormack and Shiell [13] identified a range of methods for accounting for neighbourhood self-selection, such as confounder adjustment (including the use of propensity scores) and instrumental variables. The authors noted, however, that while few cross-sectional studies adjust for neighbourhood self-selection, those studies contribute in great number to the current evidence base. However, this systematic review focussed primarily on the research findings themselves, rather than a discussion and critique of the methods for accounting for neighbourhood self-selection. To our knowledge, there have been no systematic reviews focussed on examining methods to account for neighbourhood self-selection in neighbourhood environment and physical activity research. Furthermore, there have been no reviews examining methods to deal with neighbourhood self-selection in neighbourhood environment and dietary behaviour research.

Given the potential for neighbourhood self-selection to influence estimated associations between the neighbourhood environment and both physical activity and dietary behaviours, our aims were to identify, and critique, methods used to account for and reduce the impact of self-selection on estimated associations. Specifically, our research questions were: 1) How is neighbourhood self-selection conceived in the literature on research in relation to physical activity and dietary behaviour?; 2) What methods are used to assess its impact?; and 3) amongst studies adjusting for neighbourhood self-selection (e.g., using regression based confounder adjustment), to what extent are results presented both with and without adjustment, and if so, what is the scope of variation in the study findings? Results were used to inform recommendations regarding suitable approaches for future research on neighbourhood influences on physical activity and dietary behaviour.

## Methods

### Search strategy

Search terms for neighbourhood self-selection were based on terms used in articles identified in an initial scoping review in PubMed conducted by KEL in March 2017. Search terms for physical activity were informed by those used elsewhere when examining associations between the built environment and physical activity [13]. The terms “strolling”, “leisure-time”, “recreation”, “inactivity” and “pedestrian” were excluded from the physical activity search terms to focus the search on physical activity more broadly. Search terms for diet were informed by those used elsewhere to examine associations between the food environment and diet [10]. The initial list of search terms proposed for physical activity and diet is presented in Supplementary Table 1.

Searches of these initial terms were conducted separately for each list (i.e., neighbourhood terms, self-selection terms, physical activity terms, and diet terms), as well as in combination using ‘or’ between items within each list, and ‘and’ between the different lists. Searches were conducted in Scopus, PubMed, Academic Search Complete, Education Source, ERIC, Global Health, MEDLINE Complete, SPORTDiscus, and PsycInfo through EBSCOHost on 6th July 2017 by KEL and LET. This enabled assessment of the number of articles identified using these search terms to aid in determining which terms should be included in the final search. The preliminary searches were conducted using a step by step process, first assessing the neighbourhood search terms and then adding the self-selection terms. As over 280,000 articles were identified combining the neighbourhood and self-selection terms, the search terms were reviewed to ensure relevance. This resulted in a decision to group the two neighbourhood and self-selection search terms, shown in Supplementary Table 1, together as a single neighbourhood self-selection term (e.g., “neighbo#rhood self-select*”). The final list of search terms considered in this review by database is presented in Table 1. Identified articles were transferred into EndNote. In total, 3953 articles were identified in the search, which was conducted on 19th July 2017, with 287 identified as duplicates leaving 3666 articles for screening.

**Table 1:** Search conducted for the systematic review of methods to account for self-selection in neighbourhoods and physical activity and diet research

Database	Search terms	Restrictions	Number of articles
**EBSCOHost** (Academic Search Complete, Education Source, ERIC, Global Health, MEDLINE Complete, SPORTDiscus, PsycInfo)	(“neighbo#rhood self-select*” OR “neighbo#rhood self select*” OR “neighbo#rhood select*” OR “residential self-select*” OR “residential self select*” OR “residential decisions” OR “self-selection bias” OR “self selection bias” OR “residential location decision” OR “neighbo#rhood choice” OR “neighbo#rhood preference” OR “residential mobility”) **AND** (“physical activit*” OR exercis* OR walk* OR cycl* OR bicycle* OR sport* OR “active transport*” OR “active travel” OR diet* OR nutrition* OR consumption OR food)	Adult (19+ years) English language Academic journals	1241
**PubMed**	(“neighborhood self-select*” OR “neighbourhood self-select*” OR “neighborhood self select*” OR “neighbourhood self select*” OR “neighborhood select*” OR “neighbourhood select*” OR “residential self-select*” OR “residential self select*” OR “residential decisions” OR “self-selection bias” OR “self selection bias” OR “residential location decision” OR “neighborhood choice” OR “neighbourhood choice” OR “neighborhood preference” OR “neighbourhood preference” OR “residential mobility”) **AND** (“physical activit*” OR exercis* OR walk* OR cycl* OR bicycl* OR sport* OR “active transport*” OR “active travel” OR diet* OR nutrition* OR consumption OR food)	Adult (19+ years) English language Humans	1387
**Scopus**	(“neighborhood self-select*” OR “neighbourhood self-select*” OR “neighborhood self select*” OR “neighbourhood self select*” OR “neighborhood select*” OR “neighbourhood select*” OR “residential self-select*” OR “residential self select*” OR “residential decisions” OR “self-selection bias” OR “self selection bias” OR “residential location decision” OR “neighborhood choice” OR “neighbourhood choice” OR “neighborhood preference” OR “neighbourhood preference” OR “residential mobility”) **AND** (“physical activit*” OR exercis* OR walk* OR cycl* OR bicycl* OR sport* OR “active transport*” OR “active travel” OR diet* OR nutrition* OR consumption OR food)	Adult English language Journal article Human	1325

### Screening

Two assessors independently conducted the title and abstract screening using the Rayyan app [14]. As methods for dealing with self-selection may not appear in the title or abstract, the assessors did not consider self-selection in the initial screening. To be included, articles had to: 1) be original research articles (i.e., not commentary or review articles); 2) be published as articles in refereed journals; 3) be published in English; 4) feature adult participants (i.e., ≥18 years of age); 5) include a physical activity or dietary behaviour (including food purchasing behaviours) outcome; 6) include an objectively-assessed measure of the built (e.g., food outlets, sport or recreational centres), natural (e.g., parks) or social (e.g., neighbourhood disadvantage, crime) environment as an exposure variable (articles that dealt with perceived rather than objectively assessed neighbourhood environment measures were excluded; neighbourhood environment exposure could include exposures defined for administrative units (e.g., postcode), as well as measures defined around the home address, such as buffer or proximity measures); and 7) include only community-dwelling participants (participants in hospital settings or residential care settings were excluded). Where there were disagreements, a third assessor examined the title and abstracts according to the inclusion criteria. Those that met the inclusion criteria (or, where it was not clear from the abstract that the inclusion criteria had been met) were considered in the full text assessment. Two assessors independently assessed the full text of the articles according to the same criteria applied for the title and abstract screening. A third assessment, conducted independently by two additional assessors, considered all articles that met these inclusion criteria, in addition to any where it was unclear that the criteria had been met, to determine whether self-selection had been considered.

### Data extraction

A data extraction template was created in Excel. The following information was collated from the articles included in this review: author information, article title, study setting, study design (e.g., cross-sectional, longitudinal), characteristics of participants sampled (e.g., age, gender), neighbourhood environment exposure, physical activity or diet outcome details, confounder adjustment in analyses, in addition to the method used to address neighbourhood self-selection and any limitations stated about this approach.

### Review registration

This review was registered with the PROSPERO International Prospective Register of Systematic Reviews (number CRD42018083593) and was conducted in accordance with the Preferred Reporting Items for Systematic Reviews and Meta-Analyses (PRISMA) statement.

## Results

**Fig. 2:**
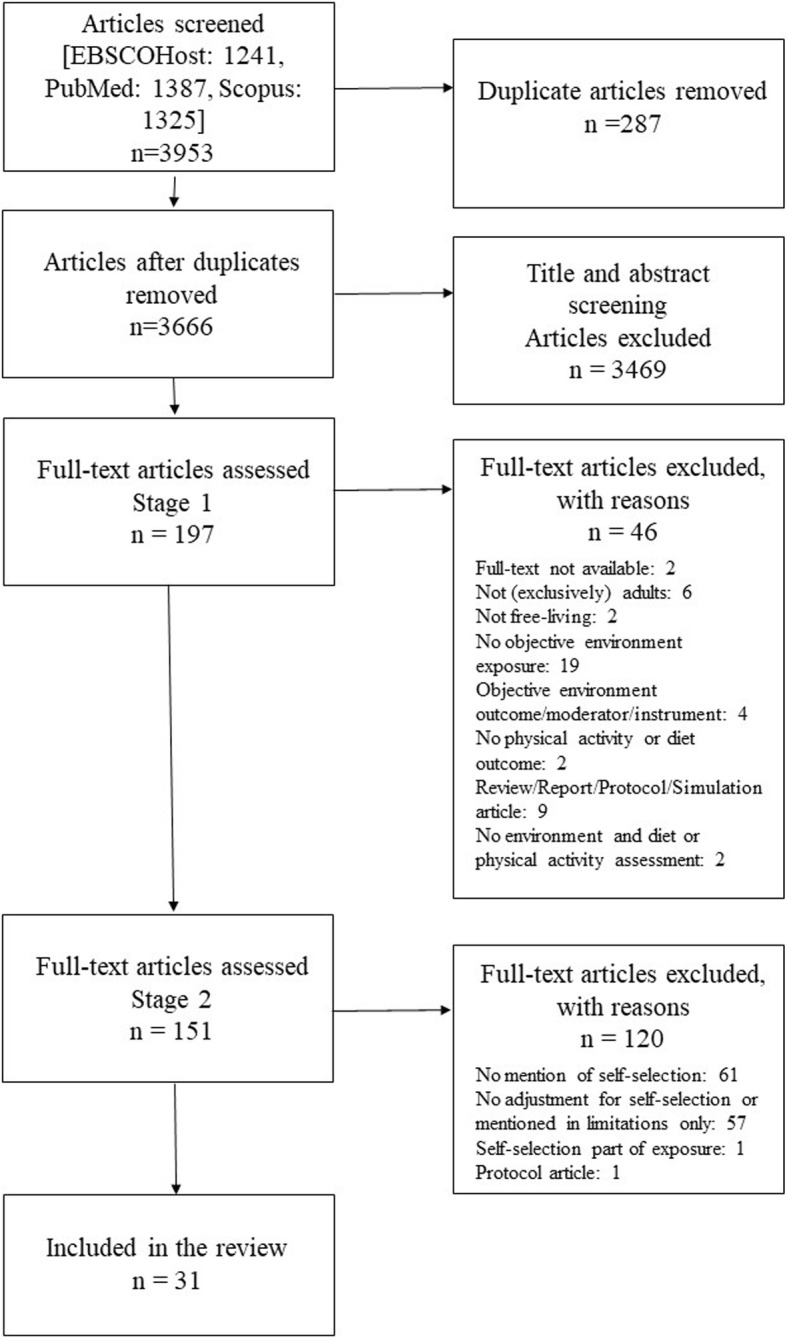
Flowchart of the systematic literature search

Of 3666 articles identified, 31 were included in the review (see Fig. 2).

A summary of included articles is presented in Table 2. Most articles reported on data from the USA (13/31) or Australia (8/31). Twenty articles featured cross-sectional analysis, 10 longitudinal analysis and one both cross-sectional and longitudinal (Table 2). Data from some studies featured in more than one article. For example, data from the Residential Environments Project (RESIDE) featured in five of the articles [22, 23, 26, 31, 34].

**Table 2:** Characteristics of the articles considered in the systematic review

Author (Year)	Country	Study name	Study design	Number of participants	Outcome	Specific outcome	Exposure	Specific exposure	Area-level exposure	Area-type	Number of areas
Alves, Silva (2013) [15]	Portugal	No name	Cross-sectional	2081	Dietary behaviour and physical activity	Fruit and vegetables, Sedentary behaviour	Social environment	Disadvantage	Yes	Census block	1662
Boarnet, Joh (2011) [16]	USA	No name	Cross-sectional	1365–1370 (depending on outcome)	Physical activity	Walking	Built environment	Residential density, Block size, Intersections, Commercial destinations	Yes	Neighbourhood	8
Boone-Heinonen, Gordon-Larsen (2011) [17]	USA	CARDIA	Longitudinal	5115	Dietary behaviour	Fast food consumption, Fruit and vegetable intake, Diet quality	Built environment	Fast food chain restaurants, Supermarkets, Smaller grocery stores	No	N/A	N/A
Boone-Heinonen, Diez Roux (2011) [18]	USA	CARDIA	Longitudinal	4179	Physical activity	Physical activity index	Social environment	Disadvantage	Yes (treated as an individual level exposure)	Census tract	Not stated
Brown, Pantin (2013) [19]	USA	No name	Cross-sectional	391	Physical activity	Walking	Built environment	Walkability	No	N/A	N/A
Brown, Lombard (2014) [20]	USA	No name	Cross-sectional	391	Physical activity	Walking	Built environment	Walkability, Distance to urban development boundary, Distance to central business district	No	N/A	N/A
Cerin, Frank (2011) [21]	USA	SMARTRAQ and NEMS	Cross-sectional	274	Physical activity	Walking	Built environment	Grocery or convenience stores, Restaurants	No	N/A	N/A
Christian, Knuiman (2013) [22]	Australia	RESIDE	Longitudinal	1047	Physical activity	Walking	Mixed	Neighbourhood type (hybrid, liveable, conventional)	No	N/A	N/A
Foster, Hooper (2016) [23]	Australia	RESIDE	Longitudinal	1813	Physical activity	Walking	Social environment	Crime	No	Suburb	Not stated
Frank, Saelens (2007) [24]	USA	SMARTRAQ	Cross-sectional	1455 and 2056 (depending on analysis)	Physical activity	Walking	Built environment	Walkability	No	N/A	N/A
Frank, Kershaw (2014) [25]	Canada	No name	Cross-sectional	2748	Physical activity	Walking	Mixed	Walkability, Household income	Yes	Forward Sortation Area (income) or Postal Code (walkability)	Not stated
Giles-Corti, Bull (2013) [26]	Australia	RESIDE	Longitudinal	1420	Physical activity	Walking	Mixed	Transport-related walking destinations (post offices, bus stops, delicatessens, supermarkets, train stations, shopping centres or CD or DVD stores), Recreation-related walking destinations (beach, park or sports field)	No	N/A	N/A
Hajna, Ross (2016) [27]	Canada	No name	Longitudinal	131	Physical activity	Daily steps	Built environment	Walkability	No	N/A	N/A
Handy, Cao (2008) [28]	USA	No name	Cross-sectional	1352 and 1497 (depending on analysis)	Physical activity	Moderate-to-vigorous physical activity	Built environment	Institutional destinations (bank, church, library, post office), Maintenance destinations (grocery store, pharmacy), Eating out destinations (bakery, pizza, ice cream, takeaway), leisure destinations (health club, bookstore, bar, theatre, video rental)	No	N/A	N/A
Jack and McCormack (2014) [29]	Canada	No name	Cross-sectional	1967	Physical activity	Walking	Built environment	Walkability	Yes	Postal codes	Not stated
Kaczynski and Mowen (2011) [30]	Canada	No name	Cross-sectional	585	Physical activity	Park based physical activity	Natural environment	Park	No	N/A	N/A
Knuiman, Christian (2014) [31]	Australia	RESIDE	Longitudinal	1703	Physical activity	Walking	Mixed	Street connectivity, Residential density, Land use mix, Service destinations (dry cleaners, post offices, pharmacies, video stores), Convenience destinations (delis, general stores, supermarkets, green grocers, seafood shops, gas stations, other food shops, shopping centres), Public open space destinations (parks, sports fields, beaches), Railway station	No	N/A	N/A
Lee, Zegras (2013) [32]	USA	No name	Cross-sectional	933	Physical activity	Walking	Mixed	Net density, Land use mix, Open space, Trail length, Intersections, Hilliness, Retail destinations, Transport destinations, Traffic volume, Traffic crashes	No	N/A	N/A
MacDonald, Stokes (2010) [33]	USA	No name	Cross-sectional and Longitudinal	498	Physical activity	Physical activity, Walking	Mixed	Light rail transit introduction [longitudinal], residential density [cross-sectional], park [cross-sectional], Food (grocery, convenience, restaurants) and alcohol destinations [cross-sectional]	No	N/A	N/A
McCormack, Shiell (2012) [34]	Australia	RESIDE	Cross-sectional	1813	Physical activity	Walking	Built environment	Street connectivity, Land-use mix, Residential density	No	N/A	N/A
McCormack, Friedenreich (2012) [35]	Canada	No name	Cross-sectional	4034	Physical activity	Walking	Mixed	Walkability, Business destinations, Bus stops, Parks, Recreational facilities, Sidewalk length, Residential density, Green space, Cycle paths	Yes	Administrative neighbourhood boundary	194
McCormack, McLaren (2017) [36]	Canada	No name	Cross-sectional	915	Physical activity	Physical activity, Walking, Cycling	Built environment	Walkability	Yes	Neighbourhood	12
Nichani, Dirks (2016) [37]	New Zealand	Growing Up in New Zealand	Cross-sectional	6772	Physical activity	Moderate-to-vigorous physical activity	Natural environment	Green space	Yes	Census area unit	413
Norman, Carlson (2013) [38]	USA	No name	Cross-sectional	240	Physical activity	Walking	Built environment	Walkability	No	N/A	N/A
Owen, Cerin (2007) [39]	Australia	PLACE	Cross-sectional	2560	Physical activity	Walking	Built environment	Walkability	Yes	Census collectors district	32
Saelens, Sallis (2012) [40]	USA	Neighbourhood Quality of Life	Longitudinal	2121	Physical activity	Moderate-to-vigorous physical activity, Walking	Mixed	Residential density, Land use mix, Intersection density, Retail destinations, Parks	No	N/A	N/A
Sallis, Saelens (2009) [41]	USA	Neighbourhood Quality of Life	Cross-sectional	2199	Physical activity	Moderate-to-vigorous physical activity, Walking	Mixed	Walkability, Household income	Yes	Census block group	32
Van Dyck, Cardon (2011) [42]	Belgium	BEPAS	Cross-sectional	412	Physical activity	Moderate-to-vigorous physical activity, Physical activity	Built environment	Walkability	Yes	Neighbourhood	24
Wells and Yang (2008) [43]	USA	No name	Longitudinal	32 and 70 (depending on analysis)	Physical activity	Walking	Built environment	Land use mix, Land use density, Street network pattern	No	N/A	N/A
West and Shores (2015) [44]	USA	No name	Longitudinal	273	Physical activity	Walking	Built environment	Greenway	No	N/A	N/A
Witten, Blakely (2012) [45]	New Zealand	URBAN	Cross-sectional	2033	Physical activity	Physical activity, Walking	Built environment	Street connectivity, Dwelling density, Land use mix, Service and amenity destinations, Urbanicity	Yes	Neighbourhood (five contiguous meshblocks)	48

*URBAN* Understanding the Relationship between Activity and Neighbourhoods, *CARDIA* Coronary Artery Risk Development in Young Adults, *PLACE* Physical Activity in Localities and Community Environments, *SMARTRAQ* Strategies for Metropolitan Atlanta’s Regional Transportation and Air Quality, *NEMS* Nutrition Environment Measures Study, *RESIDE* Residential Environments Project, *SPOTLIGHT* Sustainable Prevention of Obesity Through Integrated Strategies project, *BEPAS* Belgian Environmental Physical Activity Study

### Outcomes

A majority of studies (30/31) considered a physical activity outcome or outcomes, with walking the most commonly considered (23/30). Only two studies considered a dietary behaviour outcome [15, 17], with one of these also considering physical activity outcomes [15].

### Exposures

Nineteen articles considered individual-level environment exposure variables (e.g., total area of park space within 1 km of each participant’s home address [30]), 11 considered area-level exposure variables (e.g., percentage of green space within the census areal units in which participants reside [37]) and one article considered an area-level exposure variable but treated it as an individual-level exposure variable in the analysis as there were few individuals within each neighbourhood [18] (Table 2). Seventeen studies considered only built environment exposures (e.g., retail outlets [28]; residential density [16]; food outlets [17, 21, 28]). Two considered only natural environment exposures (parks [30]; green space [37]), while three considered only social environment exposures (socioeconomic disadvantage [15, 18]; crime [23]). Nine studies considered mixed exposure types. Fourteen studies considered a single exposure variable [15, 18, 19, 23, 24, 29, 30, 35–39, 42, 44], although one was derived from a cluster analysis of mixed environment exposure variables [35]. Walkability was the most commonly considered of these single exposure variables [19, 25, 29, 35, 36, 38, 39, 42].

### Approaches for dealing with neighbourhood self-selection

#### Model adjustment

Twenty articles used model adjustment to deal with measured neighbourhood self-selection variables (Table 3).

**Table 3:** Methods used to account for neighbourhood self-selection in the articles considered in the systematic review

Author (Year)	Study design	Outcome	Exposure	Method to account for neighbourhood self-selection	Neighbourhood self-selection variable(s)	Items/variables in derived neighbourhood self-selection variable(s)	Comparison with and without self-selection
Alves, Silva (2013) [15]	Cross-sectional	Dietary behaviour and physical activity	Disadvantage	Model adjustment	Sociodemographic characteristics	3 variables: i) Age; ii) Education; iii) Marital status	No
Boarnet, Joh (2011) [16]	Cross-sectional	Physical activity	Residential density, Block size, Intersections, Commercial destinations	Restricted population (only consider a small study area, arguing “If residential location choice mostly determines the study area where persons live, but not where along the corridor residents live, then travel behaviour differences within the corridors will be due to direct effects of differences in the built environment and business concentration, and not residential preferences.”)	N/A	N/A	No
Boone-Heinonen, Gordon-Larsen (2011) [17]	Longitudinal	Physical activity	Disadvantage	Model adjustment and Fixed effects regression (Considered both mixed and fixed effects regression)	i) Sociodemographic characteristics	5 variables: i) Education; ii) Income; iii) Race; iv) Marital status; v) Children	Yes
Boone-Heinonen, Diez Roux (2011) [18]	Longitudinal	Dietary behaviour	Fast food chain restaurants, Supermarkets, Smaller grocery stores	Fixed effects regression	N/A	N/A	No
Brown, Pantin (2013) [19]	Cross-sectional	Physical activity	Walkability	Restricted population (only considered recent Cuban immigrants who overwhelmingly reported that they did not select their neighbourhood based on built environment characteristics)	N/A	N/A	No
Brown, Lombard (2014) [20]	Cross-sectional	Physical activity	Walkability, Distance to urban development boundary, Distance to central business district	Restricted population (only considered recent Cuban immigrants who overwhelmingly reported that they did not select their neighbourhood based on built environment characteristics)	N/A	N/A	No
Cerin, Frank (2011) [21]	Cross-sectional	Physical activity	Land use mix	Restricted population (limited to middle and high-income residents who could self-select for reasons other than affordability)	N/A	N/A	No
Christian, Knuiman (2013) [22]	Longitudinal	Physical activity	Neighbourhood type (hybrid, liveable, conventional)	Model adjustment	Importance of characteristics for living in or moving to neighbourhood/new house	21 items [not provided; referenced another article]. Factor analysis identified 5 factors: i) streets are pedestrian and cycling friendly; ii) access to services, jobs or place of study; iii) access to school; iv) close to parks and recreational facilities; v) safe, diverse and easy living community.	No
Foster, Hooper (2016) [23]	Longitudinal	Physical activity	Crime	Model adjustment	Importance of characteristics for living in or moving to neighbourhood/new house	1 item: i) Importance of safety from crime	No
Frank, Saelens (2007) [24]	Cross-sectional	Physical activity	Walkability, Household income	Model adjustment	i) Importance of characteristics for living in or moving to neighbourhood/new house, ii) Neighbourhood preference	10 items in reasons for moving: i) Low crime, ii) Affordability, iii) Closeness to job, iv) Near shops and services, v) Near major roads and interstates, vi) Ease of walking, vii) Low transportation costs, viii) Near outdoor recreation, ix) Quality of schools, x) Near to public transit. Principal components analysis identified 1 factor with low transportation costs, near to public transit and ease of walking having highest loads. The average score of these three items was split into quartiles and used as the self-selection variable. 7 trade-offs used to assess preferences: i) walkability vs. commercial-residential land use separation, ii) commute distance vs. residential density, iii) urban vitality vs. low-density and single-use neighbourhoods, iv) commute distance vs. living on quieter cul-de-sac street, v) availability of alternatives to the car vs. home size, vi) accommodation of automobile vs. accommodation of pedestrians and cyclists, vii) availability of alternatives to the car vs. neighbourhood privacy. Principal components analysis identified 1 factor. This was normalised and split into quartiles.	No
Frank, Kershaw (2014) [25]	Cross-sectional	Physical activity	Walkability	Model adjustment	Neighbourhood preference	7 trade-offs used to assess preferences in walkable vs. auto-orientated neighbourhoods: i) Closeness to shops and services; ii) Level of activity and mix of housing; iii) Home size and travel options; iv) Lot size and commute distance; v) Street design and travel options; vi) Public recreation and lot size; vii) Access to and size of food stores. Trade-offs were evaluated using 11-point scales for each of three questions: 1) “Your neighbourhood preference is … “, 2) “Indicate if your current neighbourhood is more like “A” or “B” … “, 3) “Regarding [the described attributes], the neighbourhood you’d hope to find would be [more like “A” or “B”] than your current neighbourhood”. Principal component analysis was used to extract a single neighbourhood preference component. This was split into quartiles.	No
Giles-Corti, Bull (2013) [26]	Longitudinal	Physical activity	Transport-related walking destinations (post offices, bus stops, delicatessens, supermarkets, train stations, shopping centres or CD or DVD stores), Recreation-related walking destinations (beach, park or sports field)	Model adjustment	Importance of characteristics for living in or moving to neighbourhood/new house	21 items [not provided; referenced another article]. Factor analysis identified 5 factors: i) streets are pedestrian and cycling friendly; ii) access to services, jobs or place of study; iii) access to school; iv) close to parks and recreational facilities; v) safe, diverse, easy living community. These five were included as separate categorical variables (Not important or not important at all/Somewhat important/Important). In addition, a self-selection scale (not important or somewhat important) used in previous studies was considered.	Yes
Hajna, Ross (2016) [27]	Longitudinal	Physical activity	Walkability	Model adjustment	Residential self-selection	Residential self-selection: 11 items from the Neighbourhood Quality of Life Study questionnaire (reference but no details provided)	Yes
Handy, Cao (2008) [28]	Cross-sectional	Physical activity	Institutional destinations (bank, church, library, post office), Maintenance destinations (grocery store, pharmacy), Eating out destinations (bakery, pizza, ice cream, takeaway), leisure destinations (health club, bookstore, bar, theatre, video rental)	Model adjustment	Importance of characteristics when looking to move to neighbourhood/new house	34 items [not all provided]: i) Easy access to a regional shopping mall, ii) Easy access to downtown, iii) Other amenities such as a pool or community centre available nearby, iv) Shopping areas within walking distance, v) Easy access to the freeway, vi) Good public transit service (bus or rail), vii) Good bicycle routes beyond neighbourhood, viii) Sidewalks throughout neighbourhood, ix) Parks and open spaces nearby, x) Quiet neighbourhood, xi) Low crime rate within neighbourhood, xii) Low level of car traffic on neighbourhood streets, xiii) Safe neighbourhood for walking, xiv) Safe neighbourhood for kids to play outdoors, xv) Good street lighting, xvi) Diverse neighbourhoods in terms of ethnicity, race, and age, xvii) Lots of people out and about within my neighbourhood, xviii) Lots of interaction among neighbours, xix) Economic level of neighbours similar to my level, xx) Attractive appearance of neighbourhood, xxi) High level of upkeep in neighbourhood, xxii) Variety in housing styles, xxiii) Big street trees, xxiv) Large backyards, xxv) Large front yards, xxvi) Lots of off-street parking (garages or driveways). Principal components analysis identified six factors: accessibility, physical activity options, attractiveness, outdoor spaciousness, safety, and socialising.	No
Jack and McCormack (2014) [29]	Cross-sectional	Physical activity	Walkability	Model adjustment	Importance of characteristics for living in or moving to neighbourhood/new house	19 items [not provided]. Principal components analysis identified four factors: access to places that support physical activity, access to local services, sense of community, ease of driving. These were transformed into z-scores.	No
Kaczynski and Mowen (2011) [30]	Cross-sectional	Physical activity	Park	Model adjustment	Importance of characteristics for living in or moving to neighbourhood/new house	1 item: i) Importance of closeness to open space	Yes
Knuiman, Christian (2014) [31]	Longitudinal	Physical activity	Street connectivity, Residential density, Land use mix, Service destinations (dry cleaners, post offices, pharmacies, video stores), Convenience destinations (delis, general stores, supermarkets, green grocers, seafood shops, gas stations, other food shops, shopping centres), Public open space destinations (parks, sports fields, beaches), Railway station	Fixed effects regression	N/A	N/A	Yes
Lee, Zegras (2013) [32]	Cross-sectional	Physical activity	Net density, Land use mix, Open space, Trail length, Intersections, Hilliness, Retail destinations, Transport destinations, Traffic volume, Traffic crashes	Model adjustment	Self-selection	Unclear. Used structural equation modelling to enable the inclusion of latent characteristics to control for self-selection.	Yes
MacDonald, Stokes (2010) [33]	Cross-sectional and Longitudinal	Physical activity	Light rail transit introduction [longitudinal], residential density [cross-sectional], park [cross-sectional], Food (grocery, convenience, restaurants) and alcohol destinations [cross-sectional]	Propensity score and Quasi-experiment	i) Sociodemographic variables, ii) Plans to use light rail transit	Sociodemographic variables/baseline characteristics included 7 items: i) gender, ii) race, iii) age, iv) employed, v) miles to work, vi) education level, vii) rent	No
McCormack, Shiell (2012) [34]	Cross-sectional	Physical activity	Street connectivity, Land-use mix, Residential density	Propensity score	i) Importance of characteristics for living in or moving to neighbourhood/new house; ii) Number of years in current neighbourhood; iii) Sociodemographic characteristics	19 items for characteristics: i) Affordability, ii) Proximity to parks, iii) Proximity to job/school, iv) Proximity to transit, v) Proximity to stores/services, vi) Ease of walking, vii) Sense of community, viii) Safety from crime, ix) Proximity to recreation facilities, x) Access to highways, xi) Attractive streets, xii) Proximity to family/friends, xiii) Views of scenery (e.g., mountains), xiv) Cleanliness of streets, xv) Proximity to downtown, xvi) Proximity to trails, xvii) Places to be physically active, xviii) Places to walk/cycle to, xix) Ease of driving. Sociodemographic characteristics: i) home ownership status, ii) gender, iii) age, iv) education, v) number of dependents < 18 years at home	No
McCormack, Friedenreich (2012) [35]	Cross-sectional	Physical activity	Walkability	Model adjustment	Importance of characteristics for living in or moving to neighbourhood/new house	21 items [not provided; referenced another article]. Factor analysis identified 5 factors: i) Pedestrian and cycling friendly streets; ii) Accessible services for daily living; iii) Accessible schools or places of study; iv) Accessible parks and recreation facilities; 5) Housing affordability and choice. Factors included as covariates in the models.	Yes
McCormack, McLaren (2017) [36]	Cross-sectional	Physical activity	Walkability, Business destinations, Bus stops, Parks, Recreational facilities, Sidewalk length, Residential density, Green space, Cycle paths	Propensity score	Importance of characteristics for living in or moving to neighbourhood/new house	13 items: i) Proximity to transit, ii) Proximity to recreational destinations, iii) Proximity to non-recreational destinations, iv) Proximity to work, v) Proximity to schools, vi) Proximity to downtown, vii) Access to highways and major roads, viii) Access to community associations, ix) Sense of community, x) Attractiveness, xi) Cleanliness of streets, xii) Housing type variety, xiii) quality of recreational facilities. Responses to each item were collapsed from “not at all”, “somewhat” and “very important” into “not important” and “important”. Propensity scores were created.	No
Nichani, Dirks (2016) [37]	Cross-sectional	Physical activity	Green space	Model adjustment	Neighbourhood preference	1 item: “Why do you live in this neighbourhood?: I like the local lifestyle.” No/Yes. Local lifestyle included access to community resources (e.g. green space, recreational facilities, public transport, shopping, education, healthcare, social and cultural facilities).	Yes
Norman, Carlson (2013) [38]	Cross-sectional	Physical activity	Walkability	Model adjustment	i) Importance of characteristics for living in or moving to neighbourhood/new house; ii) Neighbourhood preference	4 items in moving: i) Ease of walking; ii) Near public transit; iii) Near shops and services; iv) Near outdoor recreation. The average rating of these items, all measured on 5-point scales, was split at the median to categorise as low/high importance of walkability. 3 items in preference: i) residential density; ii) land use; iii) street connectivity. The average rating of these items, all measured on 11-point scales, was split at the median to categorise as low/high preference for a high-walkability neighbourhood.	No
Owen, Cerin (2007) [39]	Cross-sectional	Physical activity	Walkability	Model adjustment	Importance of characteristics for living in or moving to neighbourhood/new house	4 items: i) Closeness to job or school; ii) Closeness to public transportation; iii) Desire for nearby shops and services; iv) Ease of walking. The average rating of these items, all on 5-point scales, was used as neighbourhood self-selection measure.	Yes
Saelens, Sallis (2012) [40]	Longitudinal	Physical activity	Residential density, Land use mix, Intersection density, Retail destinations, Parks	Model adjustment	Importance of characteristics for living in or moving to neighbourhood/new house	3 items [chosen based on factor analysis of 11 residential selection items]: i) Closeness to public transportation; ii) Desire for nearby shops and services; iii) Ease of walking. The average rating of these items, all on 5-point scales, was used as residential selection variable.	No
Sallis, Saelens (2009) [41]	Cross-sectional	Physical activity	Walkability, Household income	Model adjustment	Importance of characteristics for living in or moving to neighbourhood/new house	3 items: i) Desire for nearby shops and services; ii) Ease of walking; iii) Closeness to recreational facilities. The average rating of these items, [scale not provided but reference to paper provided] was used as measure of walkability-related self-selection of neighbourhoods.	Yes
Van Dyck, Cardon (2011) [42]	Cross-sectional	Physical activity	Walkability	Model adjustment	Importance of characteristics for living in or moving to neighbourhood/new house	21 items: i) House price; ii) Importance of living in city centre; iii) Importance of living in a quiet neighbourhood; iv-vii) Social/emotional reasons (e.g. living close to family and friends); viii-xxi) Walkability related items (e.g. importance of closeness to shops, closeness to work/school, traffic safety, amount and quality of sidewalks/footpaths). All items were scored on a 5-point scale. A single variable was created but it is not clear how this was defined. Those scoring higher than the median were considered to have walkability as an important reason for neighbourhood selection.	Yes
Wells and Yang (2008) [43]	Longitudinal	Physical activity	Land use mix, Land use density, Street network pattern	Quasi-experiment (examine post-move - pre-move change in exposure on post-move outcome controlling for pre-move outcome)	N/A	N/A	No
West and Shores (2015) [44]	Longitudinal	Physical activity	Greenway	Quasi-experiment (pre-post design with control group)	N/A	N/A	No
Witten, Blakely (2012) [45]	Cross-sectional	Physical activity	Street connectivity, Dwelling density, Land use mix, Service and amenity destinations, Urbanicity	Model adjustment	i) Sociodemographic characteristics, and ii) Neighbourhood preference	8 sociodemographic variables: i) Age; ii) Sex; iii) Ethnicity; iv) Marital status; v) Household income; vi) Educational qualifications; vii) Occupation; viii) Household car access. 2 items: i) Prefer lower-density suburban neighbourhood suburban or urban environment located 10–15 min by car from common destinations or a higher-density urban neighbourhood with most destinations accessible on foot or by public transportation within 10–15 min; ii) Strength of preference for suburban or urban environment. Responses were combined as: strongly prefer walkable, moderately prefer walkable, neutral, moderately prefer less walkable, strongly prefer less walkable.	Yes

One article, after stratifying by gender, adjusted only for age, education and marital status; these variables being framed as important confounding factors influencing neighbourhood choice [15]. These variables were typically adjusted for in the other articles included in this review, although they generally were not referred to as measures of neighbourhood self-selection. Instead, other measures relating to reasons for moving to or living in a neighbourhood were referred to as measures of neighbourhood self-selection, as described below.

Most articles that used model adjustment attempted to capture neighbourhood self-selection by measuring the importance of characteristics (such as local access to schools or recreational facilities) for living or moving to a participant’s current neighbourhood or home (11/20) [22, 23, 25, 26, 29, 30, 34, 39–42], or when looking to move (1/20) [28]. Others considered neighbourhood preference items (2/20) [37, 45], such as preference to live in an urban or suburban environment [45], or both the importance of characteristics and preference (2/20) [24, 38]. Two studies that reported using model adjustment provided little detail on the neighbourhood self-selection variables [27, 32].

#### Propensity scores

Three articles used propensity scores to deal with measured neighbourhood self-selection variables [33, 35, 36] (Table 3). McCormack et al. (2012) estimated the conditional probabilities of participants living in each of three neighbourhood clusters based on neighbourhood self-selection, in addition to the length of residence in current neighbourhood, attitude towards walking, sociodemographic characteristics, and the season the survey was conducted [35]. Nineteen self-selection items were included. A similar approach was used by McCormack et al. (2017) to examine associations between walkability (“maintainers”, “improvers”, “decliners”) and physical activity, with 13 self-selection items included [36]. MacDonald et al. (2010) considered the propensity to be in a treatment (i.e., participants in employment who used light rail transit daily) or “control” group (i.e., participants in employment who did not use light rail transit to commute to work) based on sociodemographic characteristics, social and physical environment characteristics and intention to use light rail [33]. MacDonald et al. (2010) also employed a pre-post study design to deal with neighbourhood self-selection, discussed further below.

#### Restricted population

Four articles placed restrictions on the population of participants considered, in order to deal with neighbourhood self-selection [16, 19–21]. Two articles by the same lead author [19, 20] restricted the population to recent Cuban immigrants to Florida, highlighting that this is a population “who overwhelmingly reported little choice in their selection of built environments” [20]. These studies also adjusted for age, gender and education (previously mentioned characteristics that can at least partly account for neighbourhood selection), as well as body mass index, days resident in the USA, and habitual physical activity, when examining associations between built environment characteristics, such as walkability, and physical activity [19, 20]. Another study restricted the population by only considering a small study area [16]. The fourth article restricted the population to only consider those who *could* self-select, namely those of middle and high-income whose choice was not restricted for financial reasons [21].

#### Fixed effects regression

Three articles used fixed effects regression to deal with neighbourhood self-selection in their longitudinal analyses [17, 18, 31]; one article used both this approach and mixed models with model adjustment [18]. These articles also included potential time-varying confounders in their models, such as income, marriage and children, although none included a measure of neighbourhood preference or reasons for living in current neighbourhood, such as those described in the model adjustment section.

#### Pre-post design

Three articles used a pre-post longitudinal design to deal with neighbourhood self-selection [33, 43, 44]. One study used a quasi-experimental pre-post design with a comparison group, with a 1 year follow-up after the introduction of a greenway [44]. Another article employed a pre-post design among a small subsample of participants who moved to a new neighbourhood, to examine differences in pre- versus post-move physical activity associated with a changed environment [43]. The authors asserted that self-selection was addressed as the sample consisted of women partnered with a housing program who did not have a choice between neighbourhood types as only one neighbourhood was built within each region. The third study assessed the impact of the introduction of light-rail transit on physical activity, adopting a pre-post design and utilising propensity scores, but without a comparison group [33].

### Impact of accounting for self-selection

Twelve articles compared results of analyses that accounted for neighbourhood self-selection with results of analyses that did not (Table 3). Most (10/12) had used a model adjustment approach, adjusting for measures of neighbourhood self-selection, and thus compared models with and without adjustment for self-selection variable(s) [26, 27, 30, 32, 34, 37, 39, 41, 42, 45].

Three of these 12 articles presented results from one model only, either with or without adjustment for neighbourhood self-selection, asserting in the results section that adjustment for neighbourhood self-selection had little effect on findings [27, 32], or that results were attenuated consequent to adjustment [30]. Six articles presented results for models both with and without adjustment, indicative of only small changes in coefficients or effect estimates for the exposure(s) of interest after adjustment [26, 34, 37, 39, 41, 45]. In one article examining neighbourhood walkability and physical activity, rather than present findings with and without adjustment, results were presented for the full sample of participants in addition to only those with high neighbourhood self-selection (i.e., those for whom walkability characteristics were important in their selection of neighbourhood) [42]. The results were similar in both sets of analyses.

Two articles assessed the impact of accounting for neighbourhood self-selection by comparing findings from fixed effects regression models to those from mixed models [18, 31]. Knuiman et al. (2014) highlighted that the findings from fixed effects regression were similar to those of the mixed model (and to a model fitted using generalised estimating equations which was also considered) [31]. Boone-Heinonen reported that slightly different results were produced depending on the approach taken, highlighting that confounding by unmeasured time-invariant confounders (such as neighbourhood self-selection) was minimal among black participants and stronger among white participants, but that the direction of the effect was consistent [18].

## Discussion

This review examined methods used to account for neighbourhood self-selection in studies examining associations between the built, natural and social environment on adult physical activity and dietary behaviours. In the following we provide a summary of the approaches used, an outline of the limitations of these approaches, a discussion of the variation in study findings due to accounting for neighbourhood self-selection, and an overview of other approaches that may be considered.

### Summary and limitations of approaches used to account for neighbourhood self-selection

In our review, the most common approach used to account for neighbourhood self-selection was model adjustment. The main drawback of the model adjustment approach is that this assumes that all of the important characteristics of neighbourhood self-selection are not only accurately measured and taken into account, but also that these are *valid* measures of self-selection. This may not be the case, particularly among those that were solely reliant on individual characteristics (e.g., age, sex) as proxies for neighbourhood self-selection, or those which only accounted for one aspect of neighbourhood self-selection deemed important to the exposure of interest. While individual characteristics such as age and sex may influence choice of neighbourhood, requiring accounting for in analyses, they are unlikely to be the only characteristics that influence neighbourhood choice. Analyses only adjusting for selected socio-demographic characteristics assume that *only* these characteristics influence neighbourhood selection. This ignores the fact that other sociodemographic characteristics, such as the presence of children in the household, as well as individual preferences, are likely to influence choice. Findings may be biased if *other important* characteristics that influence both the environmental exposure and the outcome are not accounted for. Therefore, it is important that researchers are cautious when identifying neighbourhood self-selection characteristics. If some of these characteristics are unmeasured, researchers should acknowledge this as a limitation of their study, noting that findings may be biased.

Regarding the validity of indicators of neighbourhood self-selection, a key challenge is the temporal ordering of variables in a causal sequence, an issue often challenging to assess given most studies are cross-sectional. For example, in a cross-sectional study examining the effect of park availability on physical activity, the importance of residing close to open space (e.g., parks) was assessed as an indicator of neighbourhood self-selection for participants’ choices for moving to their neighbourhood [30]. While this seems reasonable, it is possible that, for individuals *already* undertaking high levels of physical activity prior to moving, these existing high levels of physical activity would subsequently relate both to residing near open space and continued high levels of physical activity. Therefore, study results suggesting that greater park availability is related to physical activity behaviour, even if independent of a given preference to reside close to open space, may not wholly account for self-selection issues which should ideally be assessed temporally: retrospectively for cross-sectional studies, and prospectively for longitudinal studies.

The above example is supported by the extent of variation we uncovered in the characteristics used to account for neighbourhood self-selection. It was not obvious that any of the variables used were derived through application of an explicitly stated socio-behavioural theory. Whilst it is not the purpose of this article to explain social-behavioural theories that may apply, it should go without saying that population health research demands theoretical underpinnings [46, 47]. Theory is highly relevant to variable specification in research on environments and health, given behaviour can be explained in terms of a reciprocal interaction between cognitive, behavioural, and environmental determinants [48, 49]. A greater use of theory in variable selection would provide more support for defensible choices and, correspondingly, reduce ad hoc selection associated with large variations in choices made. This creates confusion about what should be measured to account for neighbourhood self-selection in future studies. Statistical attempts to account for neighbourhood self-selection that are devoid of theory-driven specifications of relevant measures may increase the potential for residual confounding and generate biased estimates of associations of interest. Researchers examining the effects of neighbourhood environment exposures on health-related behaviour should clearly define which attributes are most relevant and important to assess as predictors of neighbourhood self-selection.

Beyond model adjustment, propensity scores were also used to account for neighbourhood self-selection, although much less frequently than standard variable adjustment in regression models. Propensity scores are commonly used when dealing with a binary exposure (i.e., where there is an exposed and unexposed group), with a propensity score defined as the conditional probability of receiving the exposure given the observed covariates [50]. If it can be assumed that there are no unmeasured confounders (which may be infeasible – see above), then accounting for propensity scores (either via matching, stratification, as a covariate in model adjustment, or inverse-probability weighting) can enable estimation of the causal effect of the exposure. Thus, as with the model adjustment approach, propensity scores can only prevent bias from neighbourhood self-selection if all potential neighbourhood self-selection indicators are valid, measured accurately and reflected in the propensity score.

A third method used to deal with neighbourhood self-selection was to restrict the population under consideration. As argued by Brown et al. [20] and Brown et al. [19] in their studies restricted to recent immigrants, this approach is perhaps appropriate if the population considered truly does not have the ability to choose where they live. However, restriction of other populations, such as middle- and high-income groups, is unlikely to be sufficient to account for neighbourhood self-selection if the investigators do not also examine and account for the preferences that guided the particular choice of neighbourhood. Furthermore, it is questionable to assume that restriction to a particular small geographical area deals with neighbourhood self-selection, as was the case in one article considered in this review. In using this approach, Boarnet et al. (2011) argued that self-selection relates more to the area, or neighbourhood, in which people live, rather than a precise residential location in that neighbourhood [16]. However, this ignores the fact that there may be time-varying neighbourhood characteristics; such that the reasons people chose to live in, or continue to live in, a given neighbourhood may vary depending on their length of residence. For example, recent residents may have chosen to move to a particular neighbourhood due to the accessibility of resources (which may have been non-existent for earlier residents), while early residents may have moved there due to affordability of property (which may now be unaffordable for many aspiring residents). Finally, the restricted population approach is not appropriate for assessing the effect of a neighbourhood environment attribute on a health-related behaviour in the broader population, because it limits the generalisability of the findings to the particular sub-group analysed.

Longitudinal studies offer the best potential for observational studies to address how changes in the environment influence changes in behaviour. However, addressing neighbourhood self-selection prior to or during such studies remains a challenge. One approach used was to conduct within-person analyses of change using fixed effects regression which can account for any time-invariant confounding, irrespective of whether it was measured. This approach to analysing longitudinal data can be used to answer questions about how changes in an exposure affect changes in an outcome [51]. As this is a within-individual analytical approach, both measured and unmeasured time-invariant confounders such as neighbourhood self-selection, are accounted for without the need to explicitly include them in the model. This means that if the reasons for living in a neighbourhood do not change over time, these are accounted for by using this method, whether or not they were measured. Although this appears advantageous, it is perhaps unrealistic to assume that neighbourhood preference does not change over the life course. Individual preferences and reasons for choosing to reside in a particular neighbourhood may differ dependent on life stage. For example, choosing to reside near good schools may become important for those with young children, while residing in more walkable neighbourhoods may become more important as individuals age. Furthermore, fixed effects regression specifically assesses both changes in the exposure and outcome. Therefore, if there is little change in the neighbourhood environment exposure, or the outcome, over the time period of the study then fixed effects regression is not a suitable approach to adopt. As highlighted by Grafova et al. [52], this method requires longitudinal data covering a long period of time to ensure sufficient variation. Often, unfortunately, this is not available in neighbourhood and health studies [18].

Studies conducted over time can allow the use of quasi- or natural-experimental designs to assess how change influences behaviour. Of the pre-post studies considered in this review, only one had a comparator group that did not experience the change in environment. A comparison group is required to ensure that any changes over time are not attributable to another factor (or factors) unrelated to the neighbourhood environment that changed over that period. Furthermore, although a quasi-experiment enables assessing how change in an environmental characteristic shapes change in behaviour, the absence of randomisation to experimental conditions does require accounting for potential confounders in analyses. Such variables could include measures of neighbourhood self-selection. Finally, this review identified few natural experiments in this area despite recognition that such designs are important and needed to improve inference on neighbourhood effects on health [53–55]. This may be due to the practical challenges of collecting suitable pre-change data when researchers become aware of the environmental change. However, as discussed by Heinen et al., although quasi- or natural-experiments are recommended to aid in understanding the impact of neighbourhood features on behaviours, these still pose methodological challenges relating to neighbourhood self-selection. Residential relocation during the study, for example, poses a particular problem for quasi- or natural experiments and **must** be considered when undertaking analyses [56].

### Impact of adjustment for neighbourhood self-selection on findings

Although an aim of this review was to examine the scope of variation in the study findings after accounting for neighbourhood self-selection, unfortunately it was unclear what the impact of adjusting for neighbourhood self-selection was. Few studies described findings with and without accounting for self-selection. Of those studies that reported results both ways, marginal changes in the parameter estimates and thus minor changes in study conclusions, cannot be interpreted as meaning that self-selection was fully accounted for and had little impact. Providing both models can assist understanding of how the estimated effects differed dependent on adjustment, but unfortunately, as highlighted by Oakes, it is not possible to identify which neighbourhood self-selection variables are important to account for in the analysis [57]. Thus, it is not possible to argue an absence of residual confounding.

### Accounting for neighbourhood self-selection in physical activity and dietary behaviour research

It is notable that most articles identified in this review considered physical activity outcomes, with very few considering dietary behaviour outcomes. This could be because neighbourhood self-selection is simply not considered in this field, or that it is perceived to be of less importance in neighbourhood environment and dietary behaviour research than in physical activity research. However, this ignores the potential for the food environment to be an important aspect of neighbourhood selection or preference. It is possible that individuals desire to live in neighbourhoods where they have a range of food outlets. This may be particularly true of those with a preference for consuming meals out and takeaway foods. Therefore, the possibility of neighbourhood self-selection influencing associations in studies of dietary behaviour should not be overlooked. Future research is needed to examine the influence of neighbourhood self-selection in this area.

### Other approaches for dealing with neighbourhood self-selection

Although emphasised as necessary to understand neighbourhood environment effects on physical activity and dietary behaviour, it is clearly challenging to obtain longitudinal or quasi-experimental data in this field. This means that a continued predominance of cross-sectional research is likely. Unfortunately, this has major implications on our ability to establish the temporal ordering of the relationships under consideration and thus to more convincingly account for self-selection, even if applying theory to determine suitable indicators of self-selection to account for. Hence other approaches for accounting for neighbourhood self-selection suitable for use in cross-sectional research are required. Use of instrumental variables is one such promising approach, as it can deal with time-invariant as well as time-varying confounders [12]. An instrumental variable is associated with the exposure of interest and associated with the outcome only through that exposure. This technique has been used in other areas of neighbourhood environment and health research. For example, Bilger and Carrieri used neighbourhood urbanisation as an instrument when examining the association between neighbourhood problems and health [58], while Zick et al. used multiple instrumental variables (the number of churches, number of schools and proportion of the neighbourhood under 16 years of age) to examine the association between neighbourhood walkability and body mass index [59]. Although offering potential for research on neighbourhood environment and physical activity or dietary behaviour research, identifying suitable instrumental variables can be challenging as it is difficult to identify a variable that is *only* associated with the outcome through that particular environmental exposure. For example, it is likely that neighbourhood urbanisation, considered an instrumental variable by Bilger and Carrieri [58], could in addition to leading to neighbourhood problems, also lead to increased access to services, such as recreational facilities or supermarkets, which have been found to be associated with health. Therefore, any association between neighbourhood urbanisation and heath identified may not be due to neighbourhood problems as hypothesised but may be due to other alternative pathways.

### Strengths and limitations

Strengths of this review include a priori registration and reporting according to PRISMA; extensive pilot work to generate final search terms; and multiple reviewers used at each stage. The search strategy was limited to articles published in the English language and, thus, may not have included all relevant papers. By only including articles that explicitly referred to the use of methods dealing with neighbourhood self-selection, we may have missed other relevant articles which considered approaches for dealing with unmeasured confounders. Furthermore, as this review explicitly included search terms relating to neighbourhood self-selection, to identify methods used to handle this problem, the findings are not generalisable to the broader literature on objective environmental associations with physical activity and dietary behaviour. Articles that did not adjust for neighbourhood self-selection and did not mention it as a limitation did not appear in this search. In addition, this review only considered objective environmental exposures, not perceived exposures. Perceived measures become conflated with neighbourhood self-selection measures when participants report the reasons they live in particular neighbourhoods based on their perceptions of what is in their local environment (same-source bias).

Finally, like all systematic reviews, our analysis was conducted over a specific time period and developments and changes in the field that have occurred subsequently will not be reflected. While there is no strong indication in the research literature of rapid advances in methods in this space since 2017, a follow-up review should be undertaken in the future.

## Conclusions

Methods to account for neighbourhood self-selection are under-developed in research exploring links between neighbourhood environments and physical activity and dietary behaviour outcomes. Although adjusting for measured neighbourhood preference or choice attributes has the potential to reduce bias, it may be important to consider adjustment for multiple items. Future studies should consider appropriate neighbourhood self-selection measures for the environment-behaviour association under examination. Those using adjustment should: 1) provide estimates of associations with and without adjustment for neighbourhood self-selection; 2) consider temporality in the measurement of alleged self-selection variables relative to the timing of the environmental exposure and outcome behaviours in longitudinal designs; and 3) also carefully consider the theoretical plausibility of presumed pathways, and bi-directional relationships, in cross-sectional research where causal direction is impossible to establish. This will help gain a greater understanding of the impact of adjustment. Instrumental variables provide promise for dealing with neighbourhood self-selection, but these are not without their own challenges. Finally, longitudinal studies over longer periods, or quasi-experiments with appropriate comparators, may provide the most promise to understand how the neighbourhood environment influences these behaviours. However, ultimately, regardless of study design, it is recommended that future research in this field collects comprehensive information relating to neighbourhood choice and preference, as well as individual characteristics relating to these.

## Supplementary information

**Additional file 1: Table S1.** Search terms considered in initial search.

## Data Availability

All data generated or analysed during this study are included in this published article.

## Abbreviations

PRISMA: Preferred Reporting Items for Systematic Reviews and Meta-Analyses
RESIDE: Residential environments
