# Supplementary material for: Methods for accounting for neighbourhood self-selection in physical activity and dietary behaviour research: a systematic review
Source: Int J Behav Nutr Phys Act. 2020 Apr 1;17:45. doi: 10.1186/s12966-020-00947-2 (PMC7115077; doi:10.1186/s12966-020-00947-2)
Supplement: Supplementary file 1 — Additional file 1: Table S1. Search terms considered in initial search. [file 12966_2020_947_MOESM1_ESM.docx]

**SUPPLEMENTARY MATERIAL**

**Supplementary Table 1.** Search terms considered in initial search.

| **NEIGHBOURHOOD TERMS** | **SELF-SELECTION TERMS** | **BIAS TERM** | **PHYSICAL ACTIVITY** | **DIET** |
| --- | --- | --- | --- | --- |
| neighbo(u)rhood OR residen* OR communit* OR “built environment” OR location OR “physical environment” OR “social environment” | self-selec* OR choice OR preferenc* OR decision OR characteristic(s) OR selec* OR mobility OR (afford* OR pric* OR cost) | “reverse causation” OR bias OR confounding | “physical activit*” OR exercise* OR walk OR walk* OR cycl* OR bicycl* OR sport* OR sport OR “active transport” OR “active transport” OR “active travel” | consumption OR food OR diet* OR diet OR nutrition* OR nutrition |

* Indicates variations on words could be included (e.g. residence or resident or residential)
